# Spatial insights into colorectal cancer risk factors and priority areas for screening in the United Kingdom based on data from the UK Biobank

**DOI:** 10.1371/journal.pone.0328778

**Published:** 2025-07-23

**Authors:** Mei Yang, Vagheesh M. Narasimhan, Franklin Benjamin Zhan

**Affiliations:** 1 Department of Health Informatics & Information Management, Texas State University, Round Rock, Texas, United States of America; 2 Department of Geography and Environmental Studies, Texas State University, San Marcos, Texas, United States of America; 3 Department of Integrative Biology, The University of Texas at Austin, Austin, Texas, United States of America; 4 Department of Statistics and Data Science, The University of Texas at Austin, Austin, Texas, United States of America; 5 Department of Population Health, University of Texas Dell Medical School, Austin, Texas, United States of America; University of Texas School of Public Health, UNITED STATES OF AMERICA

## Abstract

Geography and geospatial data science hold the potential to make unique contributions to the reduction of the burden of cancer on society. Here we use colorectal cancer (CRC) as an example to show how spatial insights into CRC risk factors and priority areas for screening may be obtained to achieve geographically targeted screening. We obtained data from the UK Biobank and divided the participants into the older (50<=age < 70) and young (age < 50) adult groups. The data consists of 2,080 CRC cases and 8,062 controls. We used a case-control study and geographically weighted logistic regression (GWLR) to explore spatial variations in risk levels of significant factors at a fine geographic resolution. Analysis results reveal that, among all significant risk factors, polygenic risk score (PRS) is the most important risk factor for both age groups. Findings suggest that the top priority screening areas for older adults, using PRS as the sole risk factor, are between Sheffield, Birmingham, Cardiff, Bristol, and west of Greater London. For young adults, the top priority areas are between the south of Glasgow and Edinburgh and northwest of Greater London. Furthermore, the approach used in this study holds promise for developing more effective targeted cancer screening.

## Introduction

The societal burden of cancer is enormous, and this burden has been growing. Based on estimates from the International Agency for Research on Cancer [1], there were 20 million new cancer diagnosis and 9.7 million deaths worldwide in 2022 alone [1]. It is now well recognized that nearly half of cancers are preventable [2]. One effective way of preventing some cancers is screening. We advocate here that geography and geospatial data science can make unique contributions to the war against cancer in providing insights into priority geographic areas and populations for targeted screening [3]. Colorectal cancer (CRC) is one of three major cancers worldwide [1]. In this study, we use data from the UK Biobank to show how we can gain insights into the risk factors and priority areas that can be used to achieve geographically targeted CRC screening.

CRC is mostly a preventable disease, but it remains the second leading cause of cancer-related deaths in the world, with more than 900,000 deaths each year worldwide [4]. In the United Kingdom (UK), approximately 42,900 new cases are diagnosed annually [5–7], and the CRC incidence rate among young adults has increased rapidly in recent decades [8]. The incidence and mortality rates of CRC vary widely across countries, including the UK [4]. CRC screening is effective in reducing the burden of the disease [9]. An important step in developing effective CRC screening programs is the identification of risk factors associated with CRC and the delineation of priority geographic areas for targeted screening [3,10].

Previous studies explored whether CRC-associated risk factors can explain the geographic variations in CRC incidence [11,12]. Oyeyemi et al. [12] found that lifestyle-related CRC risk factors, such as smoking, education, and fruit and vegetable intake, are associated with CRC but these researchers did not explore the spatial variation of CRC incidence. Dagne [11] used Bayesian spatial models to identify clustering and hotspots of high CRC incidence rates in several Florida counties. However, this study did not examine the spatial variation of CRC-related risk factors.

Geographically weighted logistic regression (GWLR) models have been developed to explore the spatial variation of the occurrence of some diseases and possible association between these diseases and various factors. Some examples of these studies include examination of the drivers of leptospirosis [13], lung cancer risks [14], chronic obstructive pulmonary disease risk factors [15], and the presence or absence of larvae [16]. However, GWLR has hardly been used in the analysis of CRC to explore the spatial variations of its risk factors.

While genetic factors are recognized as playing an important role in CRC risk, they are rarely incorporated with other factors in spatial analyses reported in the literature. This study addresses this research gap and aims to provide spatial insights into risk factors associated with CRC and to identify priority areas for CRC screening, using genetic and other factors from the UK Biobank. In this discussion, spatial insights are geographic patterns derived from location-based observations about CRC cases in the UK and the factors that are associated with these patterns. We used a case-control study design and GWLR to identify significant risk factors associated with CRC and to explore the spatial variations in their risk levels across the UK. The overall approach and findings will contribute to the literature in targeted cancer screening and hence help reduce the burden of colorectal cancer and other cancers.

## Materials and methods

### Data source

In this study, we utilized data from the UK Biobank, consisting of participants from England, Scotland, and Wales aged 40–70 years, collected between 2006 and 2010. The variables included in the analysis were polygenic risk score (PRS), current employment status, sex, age, alcohol intake frequency, body mass index (BMI), current tobacco smoking status, index of multiple deprivation (IMD), average total household income for each individual, number of vehicles in household, maternal smoking around birth, and education level.

### Study setting and participants

This case-control study involved 10,142 White British participants who were not genetically related beyond the second degree and had no family history of CRC. Cases were selected based on ICD-10 codes of C18.0-C18.9, C19, C20, and C26.0. Controls were selected from participants in the UK Biobank who had no cancer diagnosis, were within a 5-year age range of matching CRC cases, and were living in the same output area (OA) as the CRC cases. OAs are small geographic areas created by aggregating postcode areas. The final dataset included 2,080 CRC cases and 8,062 controls.

**Fig 1:**
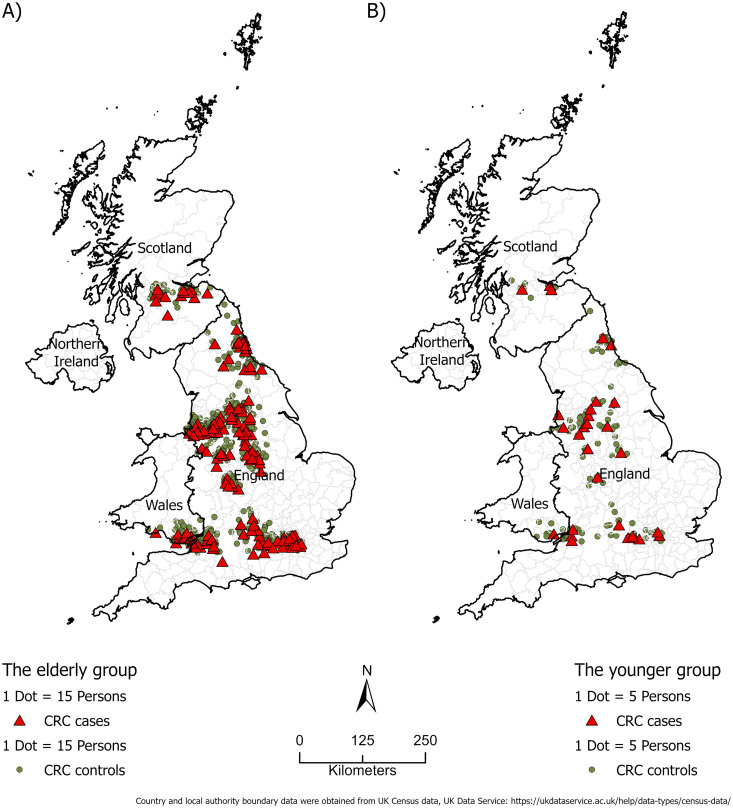
Geographic distribution of CRC cases and controls. (A) the older group (50<=age < 70). (B) the younger group (age < 50). Shown on the maps are the densities of CRC cases and controls at the local authority level. (Note: Country and local authority boundary data were obtained from UK Census data, available through the UK Data Service: https://ukdataservice.ac.uk/help/data-types/census-data/.).

**Fig 2:**
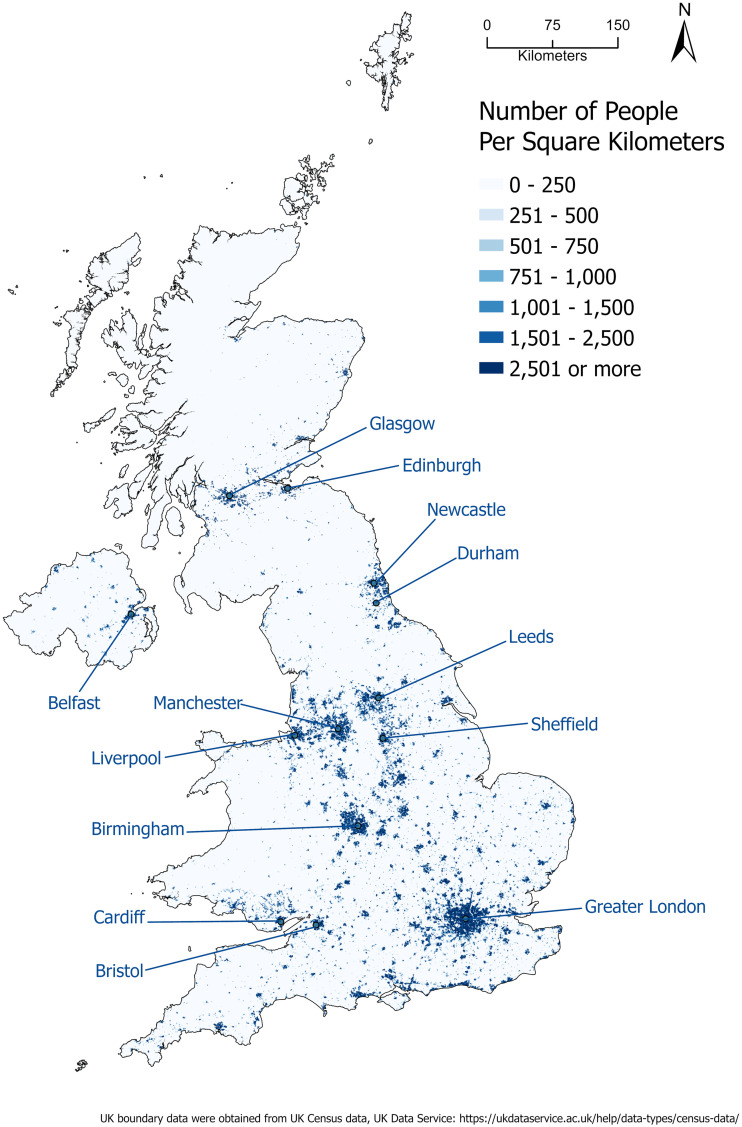
Population density in the UK. (Note: UK boundary data were obtained from UK Census data, available through the UK Data Service: https://ukdataservice.ac.uk/help/data-types/census-data/.).

We organized the cases and controls into two datasets using age 50 as the cutoff, based on prior literature [8,17]. Dataset 1 included 9,287 participants in the older group (aged 50 years or older), with 1,919 cases and 7,368 controls. Dataset 2 consisted of 855 participants in the younger group (under 50 years), with 161 cases and 694 controls. The reason for using two datasets is that the incidence of early-onset colorectal cancer (EO-CRC), defined as CRC diagnosed before age 50, has been rising at a concerning rate in recent decades among individuals younger than 50, a trend that occurs without a known etiology [8,17,18]. We wanted to examine risk factors and top priority areas for CRC screening for people younger than 50 using data from the UK Biobank. Fig 1 illustrates the geographic distribution of cases and controls for the older (Fig 1A) and the younger group (Fig 1B). Fig 2 shows the population density across the UK.

Ethics approval was not required for this analysis because the UK Biobank data is accessible to all researchers. The data was fully de-identified before we accessed it. We did not have access to any information that could identify individual participants during or after data collection.

### Polygenic risk score calculation

We calculated the PRS for each participant using the scoring function in PLINK 2.0 software [19], based on the 140 single nucleotide polymorphisms (SNPs) previously identified as risk variants for CRC among individuals of European ancestry [20]. The list of risk SNPs data and corresponding effect size on the risk of CRC can be found in the study of Thomas et al. [20]. The reason for using PRS in the analysis is that it is widely used to explore the impact of genetic risk on CRC [20,21]. Genetic data in the UK Biobank were imputed using the Haplotype Reference Consortium panel. Directly genotyped SNPs were encoded as 0, 1, or 2, representing the number of risk allele copies, while imputed SNPs were represented as dosage values, reflecting the expected number of risk allele copies. For each CRC case and control, we began by extracting all relevant risk SNPs from the imputed genotype dataset. We then calculated the PRS as the sum of risk alleles for the respective variants, using imputed dosages for imputed SNPs and 0, 1, or 2 copies of the risk allele for genotyped SNPs. Like the categorization used in the study of Jia et al. [21], we categorized the PRSs into the high PRS group (the highest 5% PRSs) and the low PRS group (the rest of the PRSs).

### Regression models

In this study, we conducted GWLR analysis to explore the spatial variations of CRC risk associated with different factors among both the older and young adult groups. GWLR is a model that can be used to effectively examine the spatial variation of relationship between dependent and independent variables at the individual level and at different spatial scales. We utilized the GWmodel package [22] in R for the GWLR analysis and used ArcGIS Pro to visualize the GWLR results on the map. Statistical significance was defined as a two-tailed p-value less than 0.05. The formula for the GWLR model is shown in Equation 1:


$$\begin{array}{c} y_{i}=\;\beta_{i0}+\sum_{k=1}^{m}\beta_{ik}x_{ik}+\epsilon_{i} \end{array}$$ (1)

Where $y_{i}$ is the dependent variable at location 𝑖; $x_{ik}$ is the value of the 𝑘^th^ independent variable at location 𝑖; *m* is the number of independent variables; $\beta_{i0}$ is the intercept parameter at location 𝑖; $\beta_{ik}$ is the local regression coefficient for the 𝑘^th^ independent variable at location 𝑖; and $\epsilon_{i}$ is the random error at location 𝑖.

The model captures the local relationships at each regression point 𝑖, with the corresponding set of regression coefficients estimated using a weighted least squares method. This estimation is represented in matrix form in Equation 2.


$${\hat{\beta }}_{i}={\left(X^{T}W\left(u_{i},v_{i}\right)X\right)}^{-1}\;X^{T}W\left(u_{i},v_{i}\right)y$$ (2)

Where *X* is the matrix of the independent variables with a column of 1s for the intercept; 𝑦 is the dependent variable vector; ${\hat{\beta }}_{i}={\left(\beta_{i0,\ldots ,\;}\;\beta_{im}\right)}^{T}$ is the vector of *m* + 1 local regression coefficient; and $W_{i}$ is the diagonal matrix denoting the geographical weighting of each observed data for regression point 𝑖 at location $\left(u_{i},v_{i}\right)$. The weight is determined by the bi-square kernel function (Equation 3), a distance-decay weighting kernel:


$$W_{ij}=\;\left\{ \begin{array}{c} {\left(1-{\left(d_{ij}/b\right)}^{2}\right)}^{2}\;if\;\left|d_{ij}\right|<b\\ 0\;otherwise \end{array}\right.\;$$ (3)

Where $W_{ij}$ is the 𝑗^th^ element of the diagonal of the matrix of geographical weights $W\;\left(u_{i},v_{i}\right)$, and $d_{ij}$ is the distance between observations 𝑖 and 𝑗, and *b* is the bandwidth. The optimal bandwidth was selected based on the smallest Akaike Information Criterion (AICc) value using the bw.ggwr function.

## Results

Table 1 lists the variables used in this study and their corresponding characteristics. For the older group, BMI values range from 15.27 to 54.52, with a median of 26.67. IMD values range from 0.82 to 81.07, with a median of 10.49. For the younger group, BMI values range from 15.84 to 53.57, with a median of 26.04. IMD values range from 1.51 to 80.29, with a median of 13.50. The older group had a significantly higher proportion of participants with daily alcohol intake (26.4% vs 17.4%) and household incomes below poverty level (20.1% vs 7.6%) compared to the younger group (Table 1).

**Table 1:** Summary information about the participants in the two datasets used in the study.

Variable	Dataset 1: Cases and Controls (50<= age < 70) (%) (1,919 cases and 7,368 controls) (N = 9,287)	Dataset 2: Cases and Controls (age < 50) (%) (161 cases and 694 controls) (N = 855)
**Age**	62 (50–70)	46 (40–49)
**Body mass index (BMI)**	26.67 (15.27–54.52)	26.04 (15.84–53.57)
**Index of multiple deprivation (IMD)**	10.49 (0.82–81.07)	13.50 (1.51–80.29)
**Sex**		
Female	4,718 (50.8)	482 (56.4)
Male	4,569 (49.2)	373 (43.6)
**Polygenic risk score (PRS)**		
Low PRS	8,851 (95.3)	809 (94.6)
High PRS	436 (4.7)	46 (5.4)
**Current tobacco smoking**		
No	8,609 (92.7)	761 (89.0)
Yes	678 (7.3)	94 (11.0)
**Alcohol intake frequency**		
Non-daily	6,838 (73.6)	706 (82.6)
Daily	2,449 (26.4)	149 (17.4)
**Household income**		
Above poverty line	7,420 (79.9)	790 (92.4)
Below poverty line	1,867 (20.1)	65 (7.6)
**Number vehicles in household**		
Have cars	8,777 (94.5)	807 (94.4)
No car	510 (5.5)	48 (5.6)
**Maternal smoking around birth**		
No	6,716 (72.3)	593 (69.4)
Yes	2,571 (27.7)	262 (30.6)
**Education**		
University	3,745 (40.3)	363 (42.5)
Non-university	5,542 (59.7)	492 (57.5)
**Employment**		
Employed	8,977 (96.7)	820 (95.9)
Unemployed	310 (3.3)	35 (4.1)

Values for continuous variables, including age, BMI, and IMD, are reported as medians, with ranges shown in parentheses. Values for categorical variables, including sex, PRS, current tobacco smoking, alcohol intake frequency, household income, number of vehicles in the household, maternal smoking around birth, education, and employment, are reported as counts, with percentages shown in parentheses.

The results of GWLR shown in Table 2 list the variables with a significant odds ratio (OR), the ranges of the values of these variables, and the significant proportion of participants associated with each of these variables in both datasets. The proportion of significant results was calculated by dividing the number of participants with statistically significant odds ratios for the risk factors (p < 0.05) by the total number of participants included in the analysis. In the older group, PRS and sex are significant CRC risk factors for all participants, with median ORs of 2.94 and 1.44, respectively. Employment is a significant CRC risk factor for 9,286 (99.9%) participants, with a median OR of 1.66. Age, BMI, and alcohol intake frequency are significant CRC risk factors for 8,579 (92.4%), 6,373 (68.6%), and 2,017 (21.7%) participants, with median ORs of 1.02, 1.01, and 1.09, respectively. In the younger group, PRS is the significant risk factor associated with CRC for all participants (median OR: 4.07). Smoking is a significant CRC risk factor for 104 (12.2%) participants with a median OR of 0.54 in the younger group.

**Table 2:** Results of geographically weighted logistic regression (GWLR) models: Factors with a significant odds ratio (OR) at the 95% significance level in each of the two datasets.

Variables	Dataset 1 GWLR OR: Cases and Controls (50<= age < 70)				Dataset 2 GWLR OR: Cases and Controls (age < 50)			
	Min	Max	Median	Significant proportion (%)	Min	Max	Median	Significant proportion (%)
Polygenic risk score (PRS)	1.97	3.31	2.94	9,287 (100)	3.93	4.13	4.07	855 (100)
Employment	1.57	1.71	1.66	9,286 (99.9)	–	–	–	–
Sex	1.40	1.52	1.44	9,287 (100)	–	–	–	–
Alcohol intake frequency	0.97	1.34	1.09	2,017 (21.7)	–	–	–	–
Age	1.01	1.03	1.02	8,579 (92.4)	–	–	–	–
Body mass index (BMI)	1.00	1.02	1.01	6,373 (68.6)	–	–	–	–
Current tobacco smoking	–	–	–	–	0.50	0.57	0.54	104 (12.2)

–, not applicable

### Spatial insights into CRC risk factors for the older group

**Fig 3:**
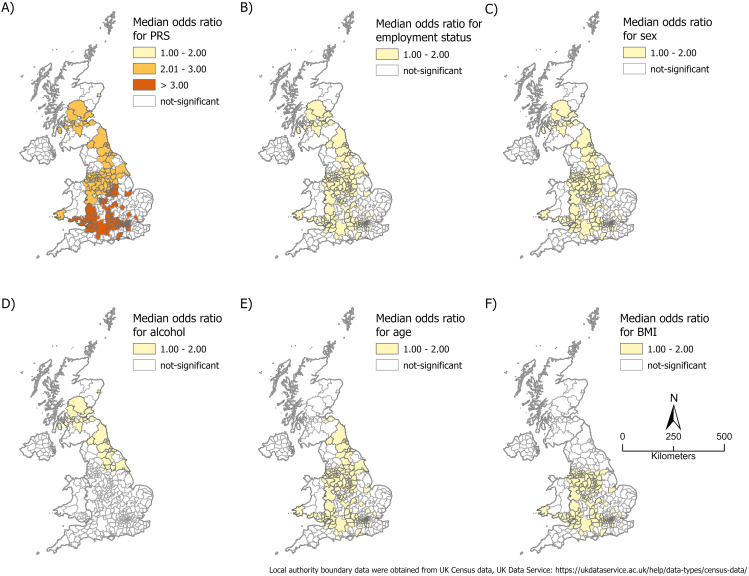
Spatial variations in CRC risk factors among older participants (50<=age < 70). (A) PRS. (B) employment status. (C) sex. (D) alcohol intake frequency. (E) age. (F) BMI. Shown on the maps are the median odds ratios in local authority’s areas. (Note: Local authority boundary data were obtained from UK Census data, available through the UK Data Service: https://ukdataservice.ac.uk/help/data-types/census-data/).

Spatial variations of different significant risk factors among participants in the older group (Dataset 1) are illustrated using choropleth maps (Fig 3). In generating the choropleth maps, we used the median odds ratio among participants in each of the local authority’s areas for each risk factor. Among all significant risk factors, PRS stands out as the risk factor with the highest odds ratios (Fig 3A). Other significant risk factors include employment status, sex, alcohol intake, age, and BMI.

There are obvious spatial variations of the level of risk for each of the significant risk factors, most noticeably PRS. Areas with the highest risk level of PRS (OR>3.0) are in the southern urban areas of the UK, including Birmingham, Cardiff, Bristol, and areas around Greater London (Fig 3A). Areas with the second highest risk level (2.0<OR<3.0) are in the central portion of the UK, including regions like Glasgow, Edinburgh, Newcastle, Durham, Manchester, Liverpool, Leeds, and Sheffield (Fig 3A).

Areas with elevated risk (1.0<OR<2.0) for employment status and sex are similar (Fig 3B and 3C). These areas are in the major urban areas of the UK, containing regions around Glasgow, Edinburgh, Newcastle, Durham, Manchester, Liverpool, Leeds, Sheffield, Birmingham, Cardiff, Bristol, and areas around Greater London (Fig 3B and 3C). Concerning alcohol, areas with elevated risk (1.0<OR<2.0) are in the central northern parts of the UK in regions around Glasgow, Edinburgh, Newcastle, and Durham (Fig 3D).

For age, areas with elevated risk (1.0<OR<2.0) are in central and southwestern parts of the UK, including areas south of Edinburgh and areas around Newcastle, Durham, Manchester, Liverpool, Leeds, Sheffield, Birmingham, Cardiff, Bristol, and the west part of Greater London (Fig 3E). Regarding BMI, areas with elevated risk (1.0<OR<2.0) are concentrated in the southwestern parts of the UK, including areas between Birmingham, Cardiff, Bristol, and the west part of Greater London (Fig 3F).

### Spatial insights into CRC risk factors for the younger group

**Fig 4:**
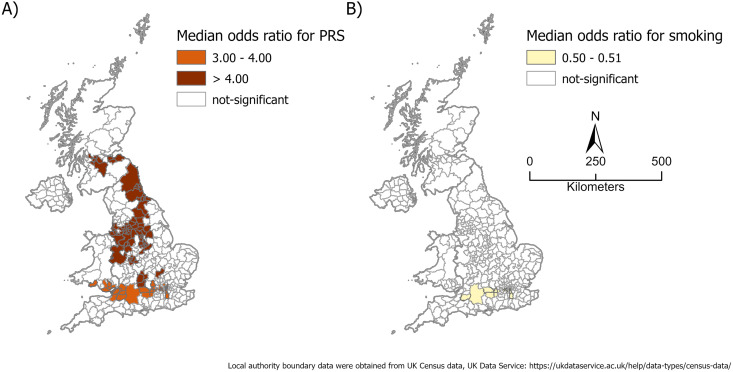
Spatial variations in CRC risk factors among younger participants (age < 50). (A) PRS. (B) smoking. Shown on the maps are the median odds ratios in local authority’s areas. (Note: Local authority boundary data were obtained from UK Census data, available through the UK Data Service: https://ukdataservice.ac.uk/help/data-types/census-data/).

The only risk factor for participants in the younger group (Dataset 2) is PRS (Fig 4A). Areas with the highest risk (OR>4.0) include urban areas between Glasgow and Edinburgh in the north, through urban areas in central parts of UK, and areas northwest of the Greater London in the south. Areas with the second highest risk (3.0<OR<4.0) are in the region between Cardiff, Bristol, and west parts of Greater London (Fig 4A). Surprisingly, smoking is a factor negatively associated with the risk of developing CRC among participants in the younger group who lived in areas between Bristol and west of Greater London with significant odds ratios varying between 0.50 and 0.51 (Fig 4B).

Overall, for participation in both the older group and the younger group, PRS is the outstanding significant risk factor for participants who lived in urban areas from Glasgow and Edinburg in the north through Cardiff, Bristol, and Greater London in the south.

## Discussion and conclusion

In this study, we aimed to examine the geographical variations of CRC risk factors for the older (50<=age < 70) and young (age < 50) adult groups in the UK based on data from the UK Biobank. Our findings revealed that: 1) polygenic risk score (PRS) is the most prominent risk factor among participants in both the older and young adult groups, 2) the spatial patterns of the level of risk associated with PRS are different between the two age groups, 3) the level of risk associated with other risk factors are less pronounced compared to that of PRS based on the values of odds ratios associated with these factors, and 4) CRC risk factors exhibit spatial variations across different regions in the UK. In addition, the overall approach used in this study provides a general framework for providing spatial insights that can be used to aid the deployment of geographically targeted cancer screening efforts.

Findings from this study suggest that: 1) PRS is potentially the most important and reliable factor for targeted screening in reducing the burden of CRC for people across all age groups in the UK, 2) geographic variations may exist among people of different age groups. It is important to keep these findings in mind when developing screening programs to prioritize screening of people of different age groups in different geographic areas. In the UK, top priority areas for people ages between 50 and 70 are areas between Birmingham, Cardiff, Bristol, and west of Greater London. For people younger than 50, the top priority areas are areas in the central part of UK between south of Glasgow and Edinburgh and northwest of Greater London.

Previous studies have widely used PRS to explore the impact of genetic risk on CRC [20,21,23–27]. PRS is essential to select screening intervals after negative findings from a colonoscopy [25]. To help define the start ages for CRC screening, Guo et al. [25] developed a polygenic risk scoring system using 90 SNPs that affect people of European descent at risk of CRC. The results indicate that people with a low and a medium PRS can have their recommended 10-year screening interval prolonged, while there is no need to shorten the screening interval for people with a high PRS.

In addition, PRS plays a crucial role in predicting and identifying high-risk individuals for CRC [20,21], as well as exploring the association between PRS and CRC risks at different stages [23,26]. Jia et al. [21] generated PRS using genome-wide association studies (GWAS) variants for eight common cancers, including CRC, based on genetic data from the UK Biobank with 400,812 participants of European descent, and found that individuals among the highest 5% of the PRS had two to threefold elevated risk for CRC. Furthermore, in the association analysis between CRC and PRS, CRC-associated common genetic variants were found to be more strongly associated with early-onset cancer than late-onset cancer [23]. These findings support the results of our study, which identified PRS as a prominent risk factor for CRC in both age groups. However, previous studies did not incorporate spatial analysis. Differing from previous studies, our study examined how to use PRS as a risk factor for prioritizing geographic areas for targeted screening. The ancestral differences between North and South Britain, with their different genetic diversities, may help explain the geographic variations in PRS risk found in this study [28].

Studies reported in the literature have shown that multiple other factors are associated with the development of CRC. Patient-level factors, such as age and gender, have been extensively studied in relation to changes in CRC incidence rates [8,29–31]. It has been found that age is associated with increased risk of CRC [30], and a greater level of CRC emergency diagnosis [29]. Males have a higher CRC risk than females [30,31]. Other published studies suggest that lifestyle factors, such as obesity (BMI > 30) [4], high alcohol intake [32], and cigarette smoking [33], are risk factors for the development of many types of cancer, including CRC. Individuals who are unemployed were less likely to participate in CRC screening compared to those who were employed [34]. These findings align with the results of our study. The spatial disparities identified in our study reflect differences in exposure to these risk factors [35].

This study is the first to analyze the spatial variation of CRC risk factors in the UK using data from the UK Biobank. The use of maps to display the geographic variations of each significant CRC risk factor provides a visually impactful method of conveying GWLR model results to healthcare providers. Moreover, the visualization of GWLR results facilitates the identification of the most critical drivers of CRC at different locations for targeted screening. A limitation in this study is that the participants were concentrated in urban regions in the UK. Our interpretation of the results has primarily focused on these urban regions, potentially overlooking the implications for rural areas. To gain a more comprehensive understanding of the broader impact of CRC risk factors, it is essential to conduct additional studies with an increased number of participants from rural regions in the UK. It should be noted that the sample size for people younger than 50 in this current study was small. This may lead to less generalizable results for this demographic. Despite the small sample size, the results of our study align with previous findings that PRS is more strongly associated with early-onset cancer than with late-onset cancer [23]. Additional research involving a larger sample size is needed to enhance the robustness and generalizability of the findings.

Future studies should further explore the concentration of significant OR for smoking in the Greater London region. In contrast to previous studies [12,36], participants with a current smoking status had less risk of CRC in the Greater London region in this study. As noted by Dimou et al. [36], spending more time smoking over a patient’s lifetime was positively associated with CRC, while patients who no longer smoked were not associated with CRC risk. Participants who were former smokers and smoked for ≥10 years or current smokers who have smoked for ≥10 years had a higher CRC risk compared to those who never smoked [12]. However, the multiple possibilities of current smokers’ status and unknown length of smoking time can be confounding factors in this study.

Furthermore, future studies should investigate the impact of population stratification on the regional variation of PRS for CRC in the UK. The impact of PRS on complex traits is known to be susceptible to population stratification [28]. The GWAS used to derive the 140 SNPs for the PRS used in this current study did not adequately control for population stratification. Additionally, ancestral differences between North and South Britain further compound this issue [28]. Therefore, uncontrolled population stratification in GWAS represents a potential confounding factor in our study. Future investigations replicating the study in diverse cohorts will help validate our findings and ensure that the results are generalizable across different population groups.

In conclusion, we used colorectal cancer (CRC) as an example and data from the UK Biobank to show how we can gain insights that may be used to prioritize CRC screening. While additional studies are needed to confirm if PRS is indeed a risk factor that can be used to facilitate CRC screening across different populations, this study has demonstrated that the overall approach holds potential to provide insights into risk factors and geographic areas to prioritize screening of CRC and other cancers.
